# Supplementary material for: Antarctic krill sequester similar amounts of carbon to key coastal blue carbon habitats
Source: Nat Commun. 2024 Sep 8;15:7842. doi: 10.1038/s41467-024-52135-6 (PMC11380667; doi:10.1038/s41467-024-52135-6)
Supplement: Supplementary file 1 — Supplementary Information [file 41467_2024_52135_MOESM1_ESM.pdf]

Antarctic krill sequester similar amounts of carbon to key coastal blue carbon habitats

Supplementary Information.

Cavan et al.

Note S1 - Abundance

KRILLBASE consists of krill density data from net hauls taken throughout the year[1], such that the time of year that sampling occurs could lead to misinterpretation/bias when looking at krill density distribution at a circumpolar scale. For instance, if a grid cell only has net haul data from April, but the adjacent grid cell has data only from January, the first April grid cell would appear to have low krill densities compared to the adjacent January one, when actually this is due to the month the water was sampled for krill. To avoid over- or under-estimating krill density in a particular area, Atkinson et al.[2] standardised the density data and present circumpolar maps based on when krill density is highest, in December and January. They convert net haul density data to what the density would be on the 1st January after fitting the regressions below with the respective number of days post October 1st, the net mouth area and regression coefficients. See also Table 4 in Atkinson et al.[1] .

D = days from October = 1, 32, 62, 93, 124, 152, 183 days
M = net mouth area (m^2) = 8 m^2
$a1$ = -0.6478
$b1$ = 2.335
$c1$ = 0.0204
$d1$ = -0.0001086
$a2$ = 0.474
$b2$ = -0.1912
$c2$ = 0.00416
$d2$ = -0.00002898

$$G1 = a1 + b1 * log10(M) + c1 * D + d1 * D^2 \qquad \text{(S1)}$$

$$G2 = 1 + exp^{(a1+b1*log10(M)+c1*D+d1*D^2)*10^{(1+b2*log10(M)+c2*D+d2*D^2)}} \qquad \text{(S2)}$$

$$Conversion\ factor = \frac{exp^{\frac{G1}{G2}}}{2.51491418861797} \qquad \text{(S3)}$$

This scales up density data to the maximum abundance likely for a particular area or cell in December/January and results in the column in KRILLBASE entitled 'STANDARDISED_ KRILL_UNDER_1M2'. Here in this study, we used this standardised density column and the conversions above to model the abundance in each cell *back* to the 1st day of each month to give estimates of krill density on a circumpolar scale and with time (Fig. S1). Our final estimates of carbon sequestration would be over-estimated if we assumed December/January abundances were relevant throughout the whole Austral spring/summer season. See also 'Abundance continued' section at end of document.

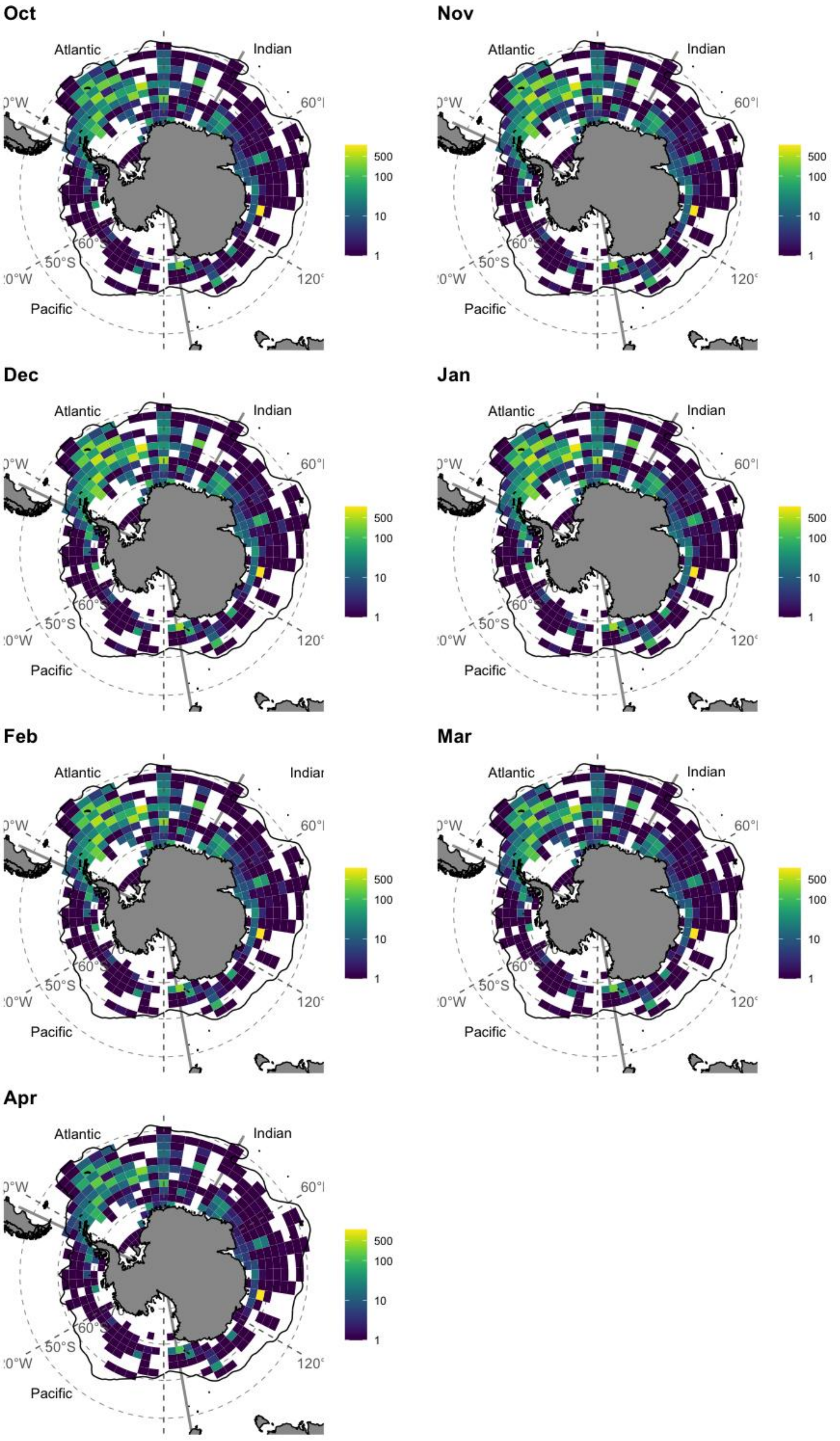

Fig. S1 Time-series krill density (# individuals m^{-2}). Density is mapped at 2° x 6° resolution applying the regression model above (Equations S1-S3) for each month.

Note S2 - Egestion rate

Our calculations require a faecal pellet egestion rate (*E*), expressed in mg C per individual per day. Published estimates such as 4.03 mg C ind^{-1} d^{-1} [3] rely on laboratory experiments where food is not limiting to the krill and represent highly productive Spring conditions only. At-sea egestion experiments over Autumn and Spring produce lower egestion rates, from 0.01-0.7 mg C ind^{-1} d^{-1} [4]. Here we explore an appropriate average egestion rate that could be applied across our sampling time series, in the absence of data on how egestion rates vary over time. We use three different approaches to estimate *E* based on 1) circumpolar krill production estimates, 2) krill daily mass growth and 3) food-web models. We provide validation for our egestion rate estimates by converting them to estimates of annual food consumption (in carbon units) by the circumpolar krill stock and comparing this to an indicative estimate of circumpolar primary production (1949 Mt C y^{-1})[5]. Our final median egestion rate of 0.46 mg C ind^{-1} d^{-1}, which we use in this study, lies within the range reported by Atkinson et al[4]. See next page.

Approach 1: estimates *E* from published estimates of circumpolar production using the equation:

$$E = \frac{P(1-AE)}{N.GGE.d} \quad \text{(S4)}$$

where *P* and *N* are estimates of a circumpolar krill production and abundance respectively, *AE* is assimilation efficiency (as a fraction), *GGE* is gross growth efficiency (as a fraction) and *d* is the number of days in the productive season. We took estimates of *P* and *N* from Atkinson *et al.*[6]. Each of these estimates is specific to an assumed individual mass of krill. We considered small (20mm = 48.4 mg wet mass), typical (40mm = 483 mg wet mass) and large (50 mm = 1127 mg wet mass) krill and converted wet mass to carbon using the conversion factor (0.1075) in Belcher *et al*[10]. We also used the *GGE* and *AE* values in Belcher *et al.*[10] and 181 days (representing the 6 months November to April). Results for each set of input values are given in Table S1.

Table S1: Assumptions and results of egestion rate calculations using Approach 1. *Q/PP* is circumpolar consumption per unit primary production where consumption (*Q*) is calculated as *P*/*GGE*.

P (Mt C)	*N* ($\times 10^{14}$)	*GGE*	*AE*	*E* (mg C d^{-1})	*Q/PP*
**Krill starting size = 20mm (5.2 mg C)**					
36.77	0.79	0.20	0.42	0.75	9%
36.77	0.79	0.20	0.75	0.32	9%
36.77	0.79	0.20	0.85	0.19	9%
36.77	0.79	0.20	0.94	0.08	9%
36.77	0.79	0.30	0.42	0.50	6%
36.77	0.79	0.30	0.75	0.22	6%
36.77	0.79	0.30	0.85	0.13	6%
36.77	0.79	0.30	0.94	0.05	6%
**mean**				0.28	8%
**Krill starting size = 40mm (40.53 mg C)**					
52.68	0.78	0.20	0.42	1.08	14%
52.68	0.78	0.20	0.75	0.47	14%
52.68	0.78	0.20	0.85	0.28	14%
52.68	0.78	0.20	0.94	0.11	14%
52.68	0.78	0.30	0.42	0.72	9%
52.68	0.78	0.30	0.75	0.31	9%
52.68	0.78	0.30	0.85	0.19	9%
52.68	0.78	0.30	0.94	0.07	9%
**mean**				0.40	11%
**Krill starting size = 50mm (94.49 mg C)**					
57.62	0.78	0.20	0.42	1.18	15%
57.62	0.78	0.20	0.75	0.51	15%
57.62	0.78	0.20	0.85	0.31	15%
57.62	0.78	0.20	0.94	0.12	15%

57.62	0.78	0.30	0.42	0.79	10%
57.62	0.78	0.30	0.75	0.34	10%
57.62	0.78	0.30	0.85	0.20	10%
**mean**				0.48	10%

Approach 2: calculates the daily egestion rate of individual krill based on observed daily mass growth:

$$E = \frac{(1-AE).m.GR}{GGE} \qquad \text{(Equation S5)}$$

where *m* is individual mean mass at the start of the growth season in mg C and *GR* is daily growth rate as a proportion of *m*. We used a *GR* estimate of 1.17% [7] alongside the values for other parameters assumed in Approach 1. Results for each set of input values are given in Table S2.

Table S2: Assumptions and results of egestion rate calculations using Approach 2. *Q/PP* is circumpolar consumption per unit primary production where consumption (*Q*) is calculated as *m.GR.d* /(*GGE.NI*) where *N* is taken from Table S1 for the relevant krill size and *d* is 181.

GR (%)	*m* (mg C)	*GGE*	*AE*	*E* (mg C d^{-1}	*Q/PP*
**Krill starting size = 20mm (5.2 mg C)**					
1.17	5.20	0.20	0.42	0.18	2%
1.17	5.20	0.20	0.75	0.08	2%
1.17	5.20	0.20	0.85	0.05	2%
1.17	5.20	0.20	0.94	0.02	2%
1.17	5.20	0.30	0.42	0.12	1%
1.17	5.20	0.30	0.75	0.05	1%
1.17	5.20	0.30	0.85	0.03	1%
1.17	5.20	0.30	0.94	0.01	1%
**mean**				0.07	2%
**Krill starting size = 40mm (40.53 mg C)**					
0.01	51.92	0.20	0.42	1.76	22%
0.01	51.92	0.20	0.75	0.76	22%
0.01	51.92	0.20	0.85	0.46	22%
0.01	51.92	0.20	0.94	0.18	22%
0.01	51.92	0.30	0.42	1.17	15%
0.01	51.92	0.30	0.75	0.51	15%
0.01	51.92	0.30	0.85	0.30	15%
0.01	51.92	0.30	0.94	0.12	15%
**mean**				0.66	18%
**Krill starting size = 50mm (94.49 mg C)**					
0.01	121.15	0.20	0.42	4.11	51%
0.01	121.15	0.20	0.75	1.77	51%
0.01	121.15	0.20	0.85	1.06	51%
0.01	121.15	0.20	0.94	0.43	51%
0.01	121.15	0.30	0.42	2.74	34%
0.01	121.15	0.30	0.75	1.18	34%
0.01	121.15	0.30	0.85	0.71	34%
0.01	121.15	0.30	0.94	0.28	34%
**mean**				1.54	43%

Approach 3: uses parameters from three regional foodweb models for habitats south of the Antarctic Polar Front compiled by Hill *et al.*[8].

$$E = \frac{(1-AE).\frac{Q}{B}.B}{\sigma.d} \qquad \text{(Equation S6)}$$

Where $\frac{Q}{B}$ is the annual carbon consumption per unit krill carbon biomass, *B,* per unit model area; σ is the number of individuals per unit model area, calculated from *B* and an assumed individual mean mass, *m*, and *d* is 181. Results for each set of input values are given in Table S3.

Table S3: Assumptions and results of egestion rate calculations using Approach 3. Model is the specific ecosystem model reported in Hill *et al.*[8] (see their Appendix A), either for South Georgia (SG), the Antarctic Peninsula (AP) and the Ross Sea (RS). *Q/PP* is circumpolar consumption per unit primary production where consumption (*Q*) is calculated as *N.Q/B.m* where *N* is taken from Table S1 for the relevant krill size and *m* is taken from Table S2 for the relevant krill size.

Model	*Q/B*	*B* (kg m^{-2})	*AE*	*E* (mg C d^{-1})	*Q/PP*
**Krill size = 20mm (5.2 mg C)**					
SG-Aggr	16.00	2.55	0.80	0.09	3%
AP-Aggr	4.70	2.34	0.73	0.04	1%
RS-Aggr	10.10	0.14	0.80	0.06	2%
**mean**				0.06	2%
**Krill starting size = 40mm (40.53 mg C)**					
SG-Aggr	16.00	2.55	0.80	0.92	33%
AP-Aggr	4.70	2.34	0.73	0.37	10%
RS-Aggr	10.10	0.14	0.80	0.58	21%
**mean**				0.62	21%
**Krill starting size = 50mm (94.49 mg C**					
SG-Aggr	16.00	2.55	0.80	2.14	78%
AP-Aggr	4.70	2.34	0.73	0.86	23%
RS-Aggr	10.10	0.14	0.80	1.35	49%
**mean**				1.45	50%

These three approaches, used with various parameter combinations, give a range of individual egestion rates spanning three orders of magnitude from 0.01 to 4.11 mg C ind^{-1} d^{-1}. Values greater than 1 mg C ind^{-1} d^{-1} occur only when either the extreme low value (0.42) is used for *AE* or krill size is assumed to be 50 mm. Values less than 0.1 mg C ind^{-1} d^{-1} occur only when either the extreme high value (0.94) is used for *AE* or krill size is assumed to be 20 mm.

There is incomplete overlap between the habitat of Antarctic krill (waters south of the Antarctic Polar Front; Atkinson et al[2]) and the area that the circumpolar primary production estimate applies to (waters south of 50°S [5]). The comparison is therefore indicative only. Consumption per unit primary production (*Q/PP*) values >30% are unlikely given that krill constitutes approximately 30% of metazoan grazer biomass in the Southern Ocean[9] and consumption by metazoans is only one of several possible fates of primary production. This comparison demonstrates that the assumption of low *AE* and/or large average krill size can lead to implausible estimates of circumpolar consumption and therefore egestion rate.

A large circumpolar KRILLBASE database of postlarval krill lengths https://doi.org/10.5285/dfbcbbf9-8673-4fef-913f-64ea7942d97a suggests that an appropriate average length is ~ 40mm [6]. Thus we use the median of egestion rate estimates for 40 mm krill (0.46 mg C d^{-1}) in our main analysis, and conduct a sensitivity analysis in the main text using the 5th and 95th percentiles of estimates for 40 mm krill (0.11 and 1.23 mg C d^{-1} respectively). Our egestion estimates are considerably lower than some values used in previous studies (e.g. Clarke et al[3] used a rate of 4.03 mg C d^{-1} and Belcher *et al.*[10] used a rate of 3.2 mg C d^{-1}). These values were based on egestion rates observed over a period of one hour which were then multiplied by 24 to give daily rates[3]. Our calculations suggest that these rates are not likely to be sustained throughout the summer season or at the circumpolar scale.

Table S4: Martin's b for krill faecal pellet POC flux adapted from Belcher et al.[10]. The median *b* is -0.30, a slight change from Belcher et al.[10] of -0.32 due to the addition of Pauli *et al.*,[16] data. We use *b* = -0.3 in this study.

Source	Region	Depth (m)	Season	Krill FP flux (mg C m^{-2} d^{-1})	Attenuation (*b*)
Belcher *et al.*[11]	South Orkneys	64	December	66.7	0.13
		165		75.5	
		76	December	33.0	1.8
		178		154.1	
		61	November	68.0	0.13
		163		77.3	
		150		*205*	
Wefer *et al.*[12] [b]	Bransfield Strait	494	January	281.2	-0.6
		1588		139.9	
Accornero *et al.*[13]	Ross sea	180	Annual mean	0.05	-0.32
		868		0.03	
Cavan *et al.*[14] [c]	Scotia Sea	70	January	58.6	0.32
		170		77.9	
González, [15] [d]	Scotia-Weddell seas	50	December-January	10	-0.63
		150		5	
		50	December-January	5.5	-2.2
		150		0.5	
		50	December-January	3	0.66
		150		10.5	
		300		9	
		50	December-January	22.5	-2.5
		150		1.5	
Pauli et al.[16]	Elephant Island	100	April	35.05	-0.61
		200		8.47	
		300		21.69	
		100	April	16.19	0.98
		200		17.7	
		300		53.24	
		100	April	25.26	-0.28
		200		11.68	
		300		20.62	
		100	April	6.76	0.44
		200		50.23	
		300		13.80	
		100	April	28.85	-0.23
		200		42.36	
		300		20.16	

[b] Fluxes are for total particulate organic carbon
[c] Fluxes are for all FP, but krill FP were dominant
[d] Fluxes are FP in terms of FP dry weight, and have been estimated from Fig. 3, Fig. 5 of González (1992)[15]

Table S5 Fraction of NPP routed to krill pellet carbon sequestration. NPP is calculated from Arteaga *et al.*[17], FPCflux is the same data as in Table 1 in the main text, and the FPCflux/NPP ratio is expressed as a percentage. Means are given with ranges in parentheses.

	Mean		
**Region**	**NPP** (mgC m^{-2} d^{-1})	**FPCflux** (mgC m^{-2} d^{-1})	**FPCflux/NPP** (%)
Atlantic	177 (90 – 352)	6.8 (0 – 120)	4.5 (0 – 58)
Indian	142 (83 – 401)	2.9 (0 – 112)	1.6 (0 – 74)
Pacific	145 (86 – 294)	1.8 (0 – 64)	0.7 (0 – 12)
All	157 (83 – 401)	4.2 (0 – 120)	2.5 (0 – 74)

Table S6 Sensitivity Analysis, see Fig. 3 in main text. Using original model parameters the total carbon sequestered from krill faeces is 19.5 MtC. Increasing krill density (abundance), egestion rate and attenuation rate of sinking pellet POC (more positive) increases MtC sequestered, whilst increasing sequestration depth decreases total MtC sequestered. Where means are given these refer to mean across whole time series, October to April and are presented in the Table for comparison across analyses, but individual grid cell values are used to calculate the new MtC values.

			**Sensitivity Analysis**			**Parameter uncertainty**		
**Parameter**	**Symbol**	**Original value**	**New value (+/- 10 %)**	**New MtC**	**Percentage change**	**New value**	**New MtC**	**Percentage change**
**Increase in parameter value**								
Density	*N*	Mean = 20 ind. m^{-2}	Mean = 22 ind. m^{-2}	21.6	110 %	Mean = 36 ind m^{-2}	35.5	180 %
Egestion	*E*	0.46 mg C d^{-1}	0.51 mg C d^{-1}	21.6	110 %	1.23 mg C d^{-1}	52.5	267 %
Sequestration depth	FPT_{100}	Mean = 381 m	Mean = 419 m	19.1	97 %	Mean = 619 m	17.0	87 %
Attenuation rate	*b*	-0.3	-0.27	21.1	108 %	+0.13	62.8	320 %
**Decrease in parameter value**								
Density	*N*	Mean = 20 ind. m^{-2}	Mean =18 ind m^{-2}	17.7	90 %	Mean = 4 ind. m^{-2}	3.9	20 %
Egestion	*E*	0.46 mg C d^{-1}	0.41 mg C d^{-1}	17.7	90 %	0.11 mg C d^{-1}	4.7	24 %
Sequestration depth	FPT_{100}	Mean = 381 m	Mean = 343 m	20.0	103 %	Mean = 187 m	24.2	123 %
Attenuation rate	*b*	-0.3	-0.33	18.1	92 %	-0.61	8.5	44 %

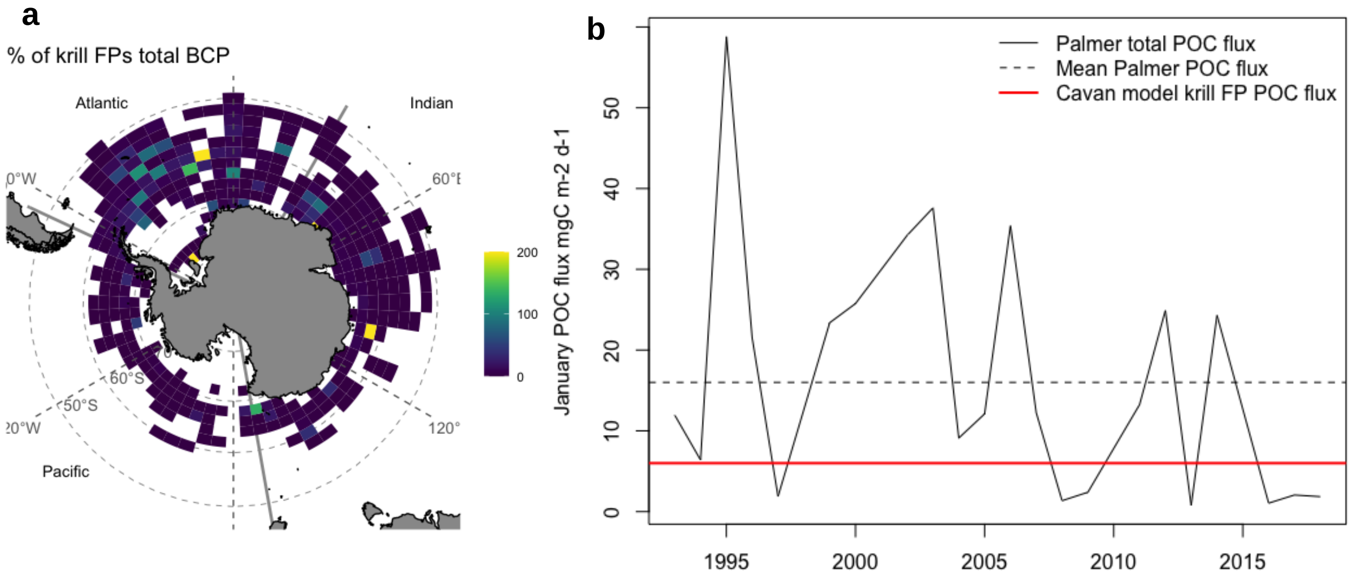

Fig S2. Krill FP sense check. a) shows the percentage contribution of krill faecal pellets to total POC flux at the sequestration depth, with the total POC flux from copepods and plankton predicted by the Nowicki *et al.*[18] model. In some locations where krill are abundant, high krill POC flux (yellow pixels) suggest the Nowicki et al. model underestimates fluxes in some regions, as their tracer model unlikely captures such high abundances of krill as KRILLBASE does and therefore does not spatially show large pulses of pellets to the ocean interior. b) shows the January sediment trap time-series data at Palmer station [19] and the average total POC flux of their January time series (dashed black line) at 170 m depth. The red line shows the average from our model of adult krill FP POC flux only, which here near the Antarctic continent represents ~ 38 % of the total flux.

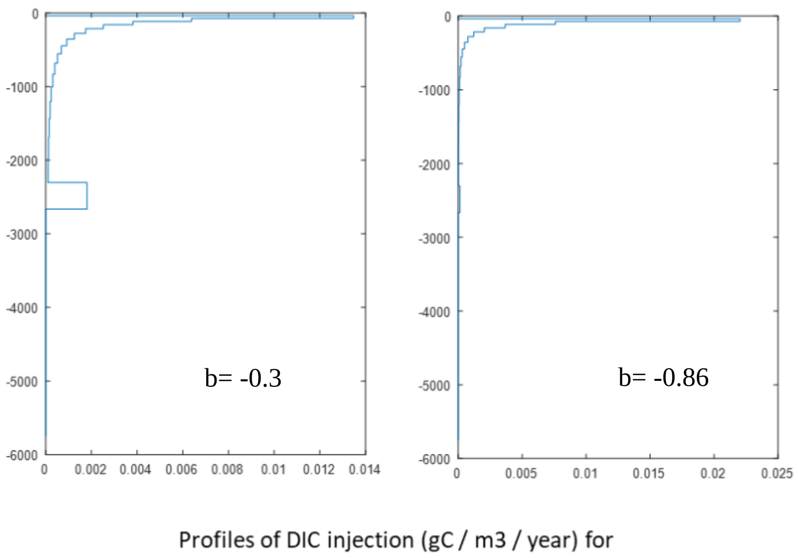

Fig S3. **Fraction of dissolved inorganic carbon (DIC) from krill pellets attenuating with depth**. Martin's b is set to -0.3 to represent observations of krill faecal pellets, and the Martin *et al.*[20] value from the equatorial Pacific, of -0.86. These data feed into the OCIM transport matrix to determine the fate of pellet-originating carbon. See Fig. 4 in the main text for when b = -0.30, and Fig. S4 below for when b = -0.86.

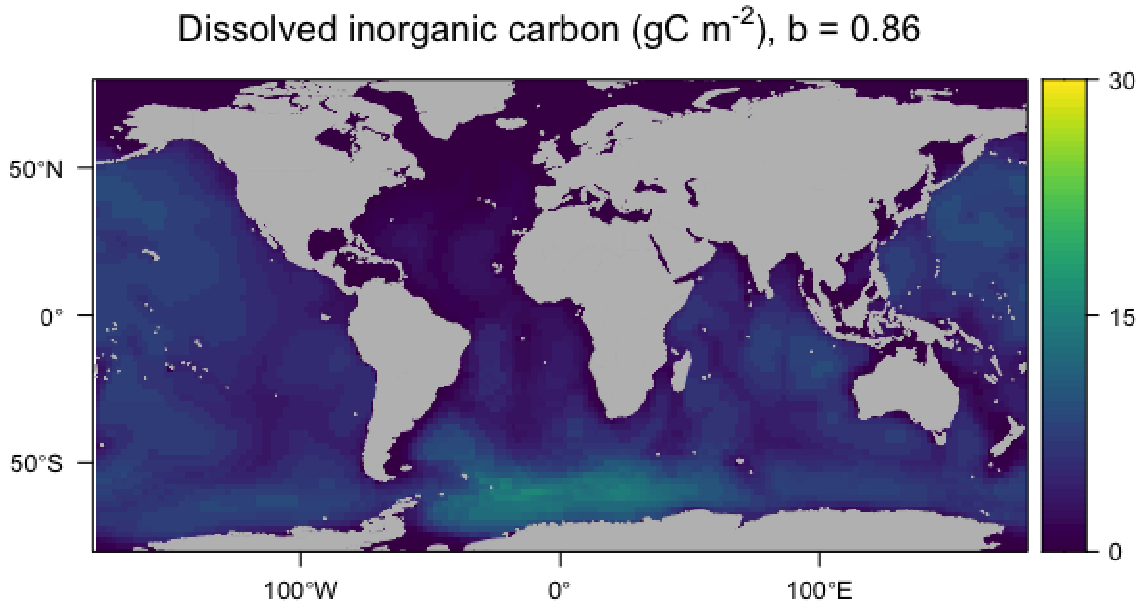

Fig S4. **Dissolved inorganic carbon (gC m^{-2}) through the water column when attenuation is set to -0.86.** The resulting carbon stored equates to 1.7 GtC for an average of 58 years.

Note S3 - Moult, carcasses and migrations

To gauge the total carbon sequestration krill may have in addition to their faecal pellets, we estimate the magnitude of other contributions from krill moults, carcasses, and vertical migrations. Moults can sink at similar rates to pellets, with sinking rates ranging from 50 to1000 m d^{-1} [21] compared to pellets which range from 27–1218 m d^{-1} [4]. The organic carbon content of moults is also high, ~ 73 % of dry mass[21]. A sediment trap near South Georgia provides monthly krill moult estimates, which throughout krill productive months (the temporal scale used in this study) equals the carbon fluxes from krill faecal pellets[22]. Therefore in Fig. 5 in the main text we suggest the magnitude of carbon fluxes from krill moults would be equal to krill pellets. For carcasses, Manno et al.[22] show that the contribution is more variable and highest in winter when krill mortality peaks. As the winter months are not included in our analysis, and due to the more limited data and knowledge on krill carcass contributions to sinking flux, we chose not to estimate circumpolar estimates in flux for carcasses.

Daily and seasonal migrations of krill can actively transfer CO_2 into the mesopelagic zone and act as efficient vectors of carbon export. To estimate this active respiratory flux we used a total circumpolar krill biomass of 380 Mt wet mass[6]. Of this total fresh mass, about 10 % is carbon (38 Mt C), with 87 % of this residing in the open ocean and the remaining 13 % living on the shelf [2]. Estimates from Schmidt *et al.*, (2011), based mainly on the summer season, suggest that ~19 % of krill in the open ocean and 2 % of krill living on the shelf reside below 400 m depth at any given time. These distributions reflect dynamic, sometimes rapidly-moving krill individuals that are migrating seasonally (Kane et al.[24]), diurnally[24,25] and at higher frequencies, partly in synchrony but likely also swimming vertically and dynamically in a non-synchronous manner in relation to individual feeding bouts [24,26]. If we assume that the biomass of krill below 400 m at any given time in shelf (~6MtC) and oceanic waters (0.1 Mt C) are respiring carbon ingested in the upper productive layers, we can do a simple calculation of active respiratory flux via vertical migration.

For this calculation, we use a representative summer daily growth rates of about 1% body C d^{-1} [7], which, based on a gross growth efficiency of 25% (i.e. growth as a percentage of ingestion [27]), would yield ingestion of 4% day, partitioned further into 1% body C d^{-1} egested and 2% body C d^{-1} respired. This fraction for respiration fits broadly within the range of measured values[28,29]. Applying a 2% body C d^{-1} respiration value for the summer months of December to March and a value one-third of this[28] for the remaining lower-food months would yield a value of 26 Mt per year respired by krill below 400m..

This estimate for the respiratory flux due to diel or higher frequency migration of krill is clearly highly uncertain. On one hand, the krill residing deeper than 400m at any one time in the summer months may be living more permanently at depth, thereby ingesting carbon at depth, possibly even at the seabed[23], and thereby not contributing to active downwards flux. On the other hand, our calculations neglect some major sources of flux due to migration. For example, the daily migrating krill may be die and sink, or be predated by deeper living fish, contributing to downwards carbon flux. In addition, the larval krill stages are not included in these calculations but are strong vertical migrators, highly abundant in strong recruitment years and

thereby likely contribute strongly in these years. Also, adult krill perform strong seasonal migrations and in winter are found at depth[24], and the respiration and mortality of these individuals contributes to flux akin to the lipid pump reported for copepods[30]. For comparison, a recent modelling study quantified the contributions of deep metazoan respiration to carbon sequestration on a large spatial scale across the sub-polar to tropical global oceans. They estimated non-polar biomass of macrozooplankton to be 80 Mt C, and the respired carbon released to be 50 Mt C, suggesting macrozooplankton respire 63 % of their total biomass[31]. Our estimates are similar, and if krill biomass is 38 MtC and they respire 26 Mt C, this is 68 % of the total biomass.

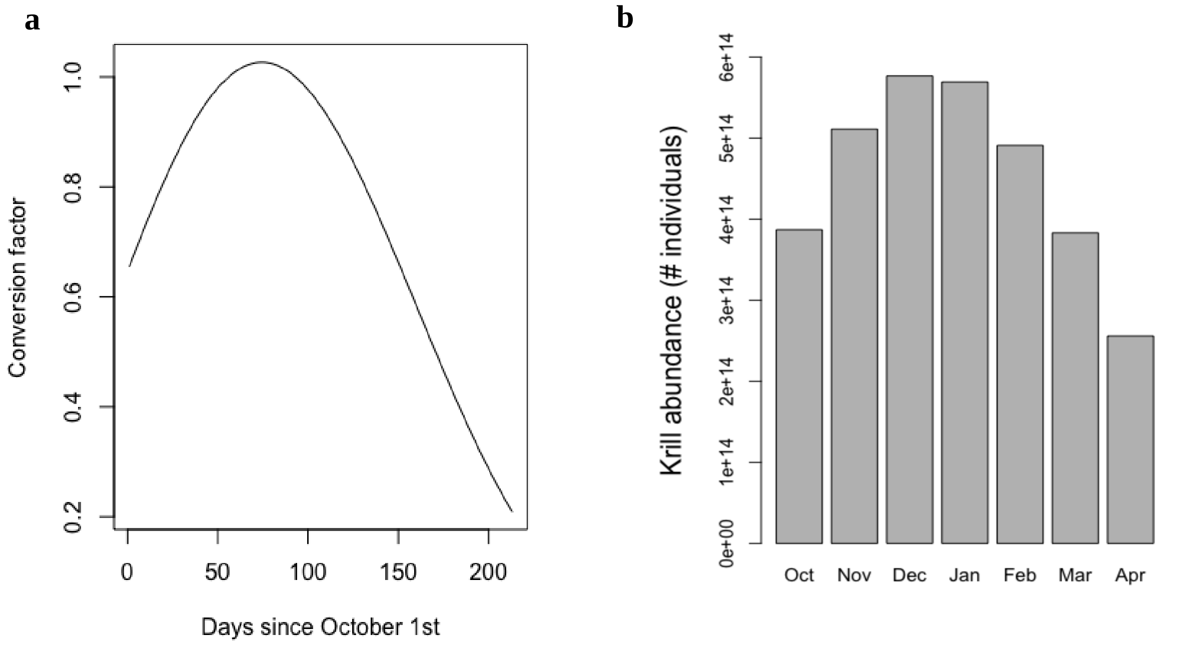

Fig. S5 Krill density modelling. Conversion factors applied to krill density (a), with a conversion of 1 in January, and slightly >1 in December. This results in circumpolar abundance estimates of krill each month (b) which are highest in December and January, and lowest in April. Note here the abundance is presented after the extreme values of > 600 ind m^{-2} are capped at 600 ind m^{-2}.

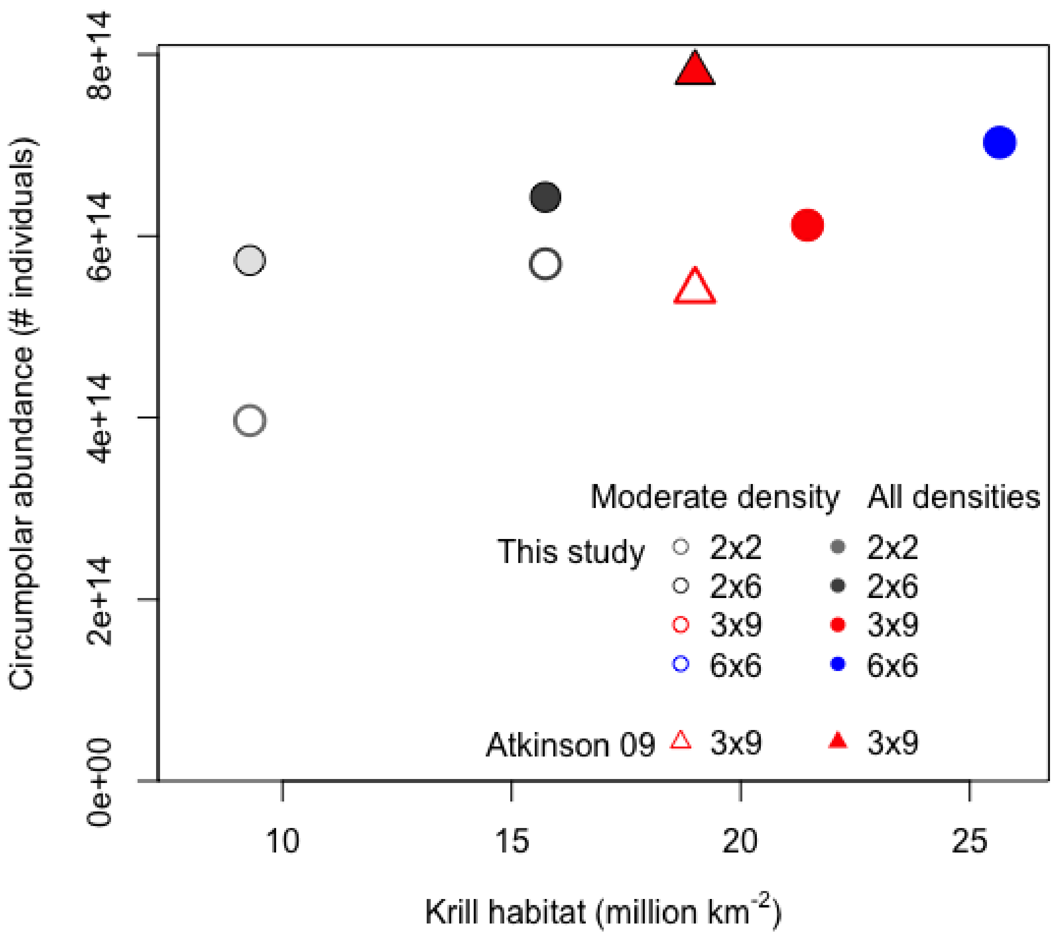

Fig. S6. Change in krill density with resolution and size of area sampled. Light grey point is at a 2°x2° resolution, dark grey is 2°x6° resolution used in this study, red point is 3°x9° resolution and blue is 6°x 6° resolution. The closed points refer to krill abundance as presented in KRILLBASE, and the open points refer to krill abundance data as in KRILLBASE however with densities >600 ind m^{-2} capped at 600 ind m^{-2}. This excludes bias from a few extremely high net catches sampling in a swarm, but still allows for high krill densities to occur (mean = 28 ind m^{-2} within the defined krill habitat, i.e. krill density > 0 ind m^{-2}, and 20 ind m^{-2} when including 0 ind m^{-2} grid cells). Open points for the 3°x9° resolution (red) and 6°x 6° resolution (blue) are the same as the closed points, as averaging over wider areas of ocean lowered the mean individual krill per m^{-2}, so that none were > 600 m^{-2}. These resolutions do increase the sampling area (Fig. S7) which directly impacts total carbon sequestered and hence we use a 2°x6° resolution. The closed red triangle is the abundance measured at 3°x9° resolution by Atkinson *et al.*,[6] using all KRILLBASE data, and the open triangle using their moderate values, where they reduced every sample >300 ind m^{-2} to their mean of 36 ind m^{-2}. The discrepancies between the closed red point and the closed red triangle in terms of krill habitat (x-axis) are likely due to different gridding approaches between the Atkinson et al.[6] paper and our study. The final circumpolar abundance we use in this study is 5.7e^{14} at a resolution of 2°x6° (dark grey open point), similar to the final abundance of 5.4e^{14} from Atkinson et al.[6] (open red triangle).

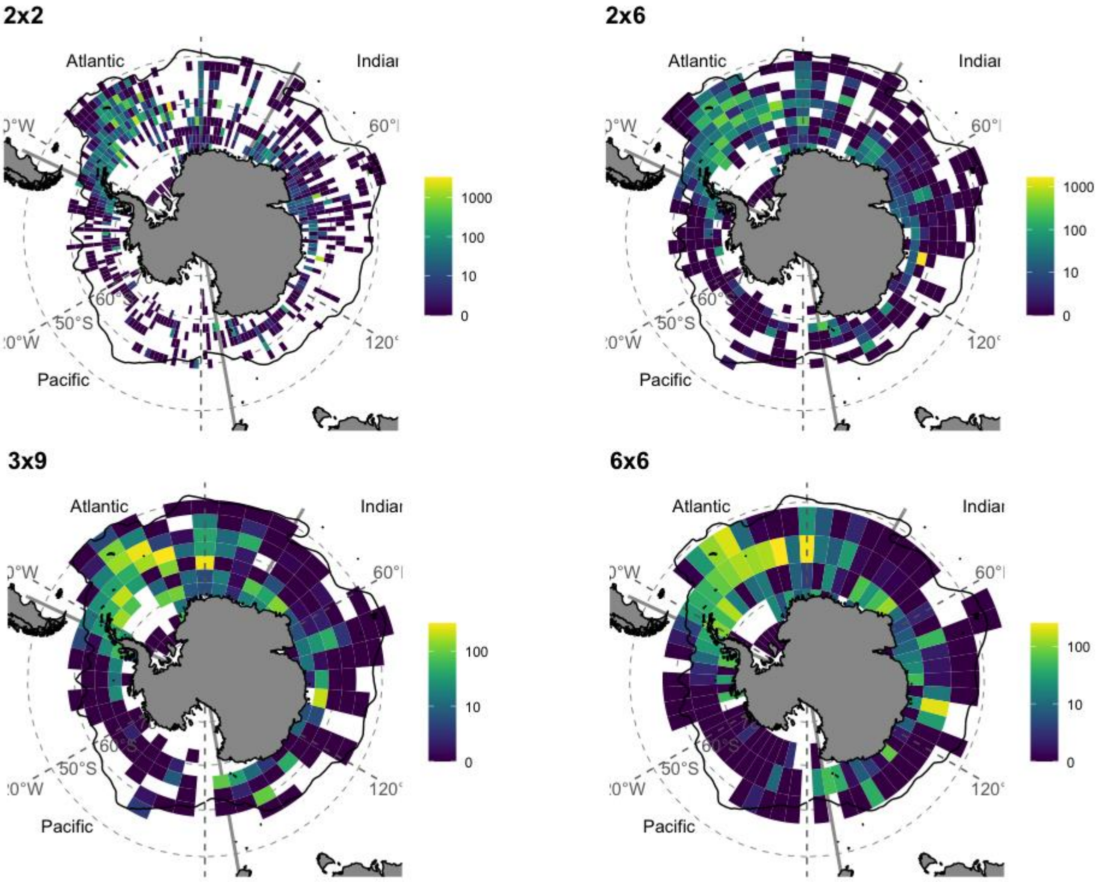

Fig. S7. **Circumpolar densities of krill at different resolutions**. Here the high densities have been retained (i.e. not capped at 600 ind m^{-2}), to show that reducing the resolution reduces the high mean abundance values in each grid cell, whilst increasing the habitat area. Consequently mean density within grid cells tends to decrease as habitat area coverage increases, which explains the absence of a linear trend in Fig. S6.

<u>Supplementary References</u>

1. Atkinson, A. *et al.* KRILLBASE: A circumpolar database of Antarctic krill and salp numerical densities, 1926-2016. *Earth Syst. Sci. Data* **9**, 193–210 (2017).
2. Atkinson, A. *et al.* Oceanic circumpolar habitats of Antarctic krill. *Mar. Ecol. Prog. Ser.* **362**, 1–23 (2008).
3. Clarke, A., Quetin, L. B. & Ross, R. M. Laboratory and field estimates of the rate of faecal pellet production by Antarctic krill, Euphausia superba. *Mar. Biol.* **98**, 557–563 (1988).
4. Atkinson, A., Schmidt, K., Fielding, S., Kawaguchi, S. & Geissler, P. A. Variable food absorption by Antarctic krill: Relationships between diet, egestion rate and the composition and sinking rates of their fecal pellets. *Deep. Res. Part II Top. Stud. Oceanogr.* **59**–**60**, 147–158 (2012).
5. Arrigo, K. R., van Dijken, G. L. & Bushinsky, S. Primary production in the Southern Ocean, 1997–2006. *J. Geophys. Res. Ocean.* **113**, (2008).
6. Atkinson, A., Siegel, V., Pakhomov, E. A., Jessopp, M. J. & Loeb, V. A re-appraisal of the total biomass and annual production of Antarctic krill. *Deep. Res. Part I*

Oceanogr. Res. Pap. **56**, 727–740 (2009).
7. Atkinson, A. *et al.* Natural growth rates in Antarctic krill (Euphausia superba): II. Predictive models based on food, temperature, body length, sex, and maturity stage. *Limnol. Oceanogr.* **51**, 973–987 (2006).
8. Hill, S. L. *et al.* Robust model-based indicators of regional differences in food-web structure in the Southern Ocean. *J. Mar. Syst.* **220**, 103556 (2021).
9. Yang, G., Atkinson, A., Pakhomov, E. A., Hill, S. L. & Racault, M.-F. Massive circumpolar biomass of Southern Ocean zooplankton: Implications for food web structure, carbon export, and marine spatial planning. *Limnol. Oceanogr.* **n/a**, (2022).
10. Belcher, A. *et al.* Krill faecal pellets drive hidden pulses of particulate organic carbon in the marginal ice zone. *Nat. Commun.* **10**, (2019).
11. Belcher, A. *et al.* The potential role of Antarctic krill faecal pellets in efficient carbon export at the marginal ice zone of the South Orkney Islands in spring. *Polar Biol.* **40**, 2001–2013 (2017).
12. Wefer, G., Fischer, G., Fuetterer, D. & Gersonde, R. Seasonal particle flux in the Bransfieid Strait, Antarctica. *Deep Sea Res.* **35**, 891–898 (1988).
13. Accornero, A., Manno, C., Esposito, F. & Gambi, M. C. The vertical flux of particulate matter in the polynya of Terra Nova Bay . Part II . Biological components. *Antarct. Sci.* **15**, 175–188 (2003).
14. Cavan, E. L. *et al.* Attenuation of particulate organic carbon flux in the Scotia Sea, Southern Ocean, is controlled by zooplankton fecal pellets. *Geophys. Res. Lett.* **42**, 821–830 (2015).
15. González, H. E. The distribution and abundance of krill faecal material and oval pellets in the Scotia and Weddell Seas (Antarctica) and their role in particle flux. *Polar Biol.* **12**, 81–91 (1992).
16. Pauli, N.-C. *et al.* Krill and salp faecal pellets contribute equally to the carbon flux at the Antarctic Peninsula. *Nat. Commun.* **12**, 7168 (2021).
17. Arteaga, L., Haëntjens, N., Boss, E., Johnson, K. S. & Sarmiento, J. L. Assessment of Export Efficiency Equations in the Southern Ocean Applied to Satellite-Based Net Primary Production. *J. Geophys. Res. Ocean.* (2018). doi:10.1002/2018JC013787
18. Nowicki, M., DeVries, T. & Siegel, D. A. Quantifying the Carbon Export and Sequestration Pathways of the Ocean's Biological Carbon Pump. *Global Biogeochem. Cycles* **36**, e2021GB007083 (2022).
19. Trinh, R., Ducklow, H. W., Steinberg, D. K. & Fraser, W. R. Krill body size drives particulate organic carbon export in West Antarctica. *Nature* **618**, 526–530 (2023).
20. Martin, J. H., Knauer, G. A., Karl, D. M. & Broenkow, W. W. VERTEX: carbon cycling in the northeast Pacific. *Deep Sea Res. Part I Oceanogr. Res. Pap.* **34**, 267–285 (1987).
21. Nicol, S. & Stolp, M. Sinking rates of cast exoskeletons of Antarctic krill (Euphausia superba Dana) and their role in the vertical flux of particulate matter and fluoride in the Southern Ocean. *Deep Sea Res. Part A. Oceanogr. Res. Pap.* **36**, 1753–1762 (1989).
22. Manno, C., Fielding, S., Stowasser, G., Murphy, E. J. & Thorpe, S. E. Continuous moulting by Antarctic krill drives major. *Nat. Commun.* (2020). doi:10.1038/s41467-020-19956-7
23. Schmidt, K. *et al.* Seabed foraging by Antarctic krill: Implications for stock assessment, bentho-pelagic coupling, and the vertical transfer of iron. *Limnol. Oceanogr.* **56**, 1411–1428 (2011).
24. Kane, M. K., Yopak, R., Roman, C. & Menden-Deuer, S. Krill motion in the Southern Ocean: quantifying in situ krill movement behaviors and distributions during the late

austral autumn and spring. *Limnol. Oceanogr.* **63**, 2839–2857 (2018).
25. Hernández-León, S., Portillo-Hahnefeld, A., Almeida, C., Bécognée, P. & Moreno, I. Diel feeding behaviour of krill in the Gerlache Strait, Antarctica. *Mar. Ecol. Prog. Ser.* **223**, 235–242 (2001).
26. Tarling, G. A. & Thorpe, S. E. Oceanic swarms of antarctic krill perform satiation sinking. *Proc. R. Soc. B Biol. Sci.* **284**, (2017).
27. Straile, D. Gross growth efficiencies of protozoan and metazoan zooplankton and their dependence on food concentration, predator-prey weight ratio, and taxonomic group. *Limnol. Oceanogr.* **42**, 1375–1385 (1997).
28. Meyer, B. & Teschke, M. Physiology of Euphausia superba. *Biol. Ecol. Antarct. krill* 145–174 (2016).
29. Bernard, K. S., Steinke, K. B. & Fontana, J. M. Winter condition, physiology, and growth potential of juvenile Antarctic krill. *Front. Mar. Sci.* **9**, 990853 (2022).
30. Jónasdóttir, S. H., Visser, A. W., Richardson, K. & Heath, M. R. Seasonal copepod lipid pump promotes carbon sequestration in the deep North Atlantic. *Proc. Natl. Acad. Sci.* **112**, 12122–12126 (2015).
31. Pinti, J. *et al.* Model estimates of metazoans' contributions to the biological carbon pump. *Biogeosciences* **20**, 997–1009 (2023).
